# Targeting PARP1 to Enhance Anticancer Checkpoint Immunotherapy Response: Rationale and Clinical Implications

**DOI:** 10.3389/fimmu.2022.816642

**Published:** 2022-04-27

**Authors:** Carlos Wagner S. Wanderley, Tatiana Strava Correa, Mariana Scaranti, Fernando Queiroz Cunha, Romualdo Barroso-Sousa

**Affiliations:** ^1^Center for Research in Inflammatory Diseases (CRID), Ribeirao Preto Medical School, Ribeirao Preto, Brazil; ^2^Department of Pharmacology, Ribeirao Preto Medical School, University of São Paulo, Ribeirao Preto, Brazil; ^3^Oncology Center, Hospital Sírio-Libanês, Brasília, Brazil; ^4^Oncologia DASA, Hospital Nove de Julho, São Paulo, Brazil

**Keywords:** cancer, immunotherapy, DNA damage, immune response, PARP (poly(ADP-ribose), polymerase

## Abstract

Reinvigorating the antitumor immune response using immune checkpoint inhibitors (ICIs) has revolutionized the treatment of several malignancies. However, extended use of ICIs has resulted in a cancer-specific response. In tumors considered to be less immunogenic, the response rates were low or null. To overcome resistance and improve the beneficial effects of ICIs, novel strategies focused on ICI-combined therapies have been tested. In particular, poly ADP-ribose polymerase inhibitors (PARPi) are a class of agents with potential for ICI combined therapy. PARPi impairs single-strand break DNA repair; this mechanism involves synthetic lethality in tumor cells with deficient homologous recombination. More recently, novel evidence indicated that PAPRi has the potential to modulate the antitumor immune response by activating antigen-presenting cells, infiltrating effector lymphocytes, and upregulating programmed death ligand-1 in tumors. This review covers the current advances in the immune effects of PARPi, explores the potential rationale for combined therapy with ICIs, and discusses ongoing clinical trials.

## Introduction

Cancer immunotherapy has revolutionized the field of oncology by demonstrating that the use of immune checkpoint inhibitors (ICIs) alone or in combination with other therapies prolongs the survival of patients with advanced disease, including melanoma, genitourinary, lung, gastric, and more recently breast cancer (1–4). However, the efficacy of ICIs varies depending on the type of cancer and within the same tumor tissue cohort (5). Ultimately, the benefits of ICI therapy in the overall population could be considered low, especially in some common tumor types, such as prostate and breast cancers (6, 7).

In this context, strategies to enhance the benefit of ICIs have focused on patient selection based on biomarkers such as programmed death ligand-1 (PD-L1) or the use of ICIs combined with other agents, including chemotherapy or targeted therapy (4, 5).

Poly (ADP-ribose) polymerase (PARP) inhibitors (PARPi) are a class of drugs that inhibit single-strand DNA repair, leading to DNA damage and apoptosis (8). Notably, this process of DNA damage can modulate the antitumor immune response by activating antigen-presenting cells (APCs), infiltrating effector lymphocytes, and upregulating PD-L1 in tumors. In this review, we summarize the current knowledge on the immune-mediated effects of PARPi and the rationale for clinical trials that combine these agents with ICIs.

## DNA Damage Repair Pathways and Cancer

The genome of every cell is constantly exposed to endogenous and/or exogenous sources of DNA damage. Usually, a chemical addition or disruption to a base of DNA or a break in one or both chains of DNA strands is characterized as DNA damage (9). DNA damage mechanisms for detecting and repairing DNA, collectively termed DNA damage response (DDR), are activated to ensure cell survival (10). Thus, dysregulation and mutations in these DDR factors and their modulators have implications for human health and disease, including increased susceptibility to DNA mutations that can lead to neoplastic transformation (9, 10). High levels of replication stress often induce DNA damage in cancer cells and their survival relies on certain DNA repair pathways (11). Understanding the broader role of DDR pathways in cancers has led to the development of pharmacological interventions for cancer therapy, such as drugs targeting poly (ADP-ribose) polymerases (PARP) (12).

### DNA Repair and PARP

PARP belongs to a family of 17 enzymes involved in several cellular processes, including DDR (13). Poly (ADP-ribose) polymerase 1 (PARP1), the most well-known enzyme in this family, is involved in the detection and repair of DNA single-strand breaks (13, 14). Functionally, PARP1 can rapidly detect DNA damage. The binding of PARP1 to DNA alters its catalytic domains, causing PARP1 to catalyze the post-translational polymerization of ADP-ribose units (15). PARP1 enables the auto-PARylation and PARylation of histones and other chromatin-associated proteins. Finally, PARP1 recruits additional DNA repair molecules, such as X-ray repair cross complementing 1 (XRCC1), to the site of damage, promoting the effective repair of DNA (8, 16, 17). However, when PARP fails or is pharmacologically inhibited, single-stranded breaks accumulate and become double-stranded breaks (18). Cells with an increasing number of double-strand breaks become more dependent on other repair pathways, mainly homologous recombination (HR) and non-homologous end joining (NHEJ) (19). The two main pathways involved in DNA double-strand break repair are described below:

### Homologous Recombination (HR)

HR is an efficient and high-fidelity DNA repair mechanism based on a homologous template (8, 20). The HR pathway mainly occurs during the S/G2 phase of the cell cycle (21). HR is initiated by the MRN-complex, composed of meiotic recombination 11 (MRE11), RAD50 homolog (RAD50), and Nijmegen breakage syndrome 1 (NBS1), which is recruited to the sites of double-strand breaks (22). The MRN complex produces a 3 overhang of single-stranded DNA that is coated by replication protein A (RPA) to avoid DNA secondary structure formation (8, 20). Breast cancer susceptibility genes 1 and 2 (*BRCA1* and *BRCA2*) enable DNA repair protein RAD51 homolog 1 (RAD51) recombinase to displace RPA and stabilize RAD51-single-stranded DNA filaments (20, 23). These filaments invade a sister chromatid to execute the homology search, and repair-associated DNA synthesis is terminated by the generation of a double-Holliday junction, which leads to the effective repair of the DNA double-strand break (21). Therefore, tumor cells with defective HR, such as those with a *BRCA1/2* mutation, are susceptible to impairment of PARP, facilitating cell death, or can be alternatively repaired by the error-prone NHEJ pathway, resulting in genomic instability before cell death (24, 25).

### Non-Homologous End Joining (NHEJ)

NHEJ repairs double-strand breaks in DNA without a template strand. Consequently, NHEJ is an error-prone double-strand break repair mechanism. It does not require a template strand and can be activated in all phases of the cell cycle (8, 26). The initial step in NHEJ is recognition and binding of the Ku heterodimer protein (Ku70/80) to double-strand breaks. The Ku-DNA complex acts as a scaffold for DNA-dependent protein kinase (DNA-PKcs) and enzymes such as X-ray cross-complementing protein 4 (XRCC4), XRCC4-like factor (XLF), and DNA ligase IV, which ligate DNA and mediate the ligation of the double-strand break (8, 26). In this context, PARPi in HR-deficient cells promotes NHEJ DNA repair and induces genomic instability or cell death.

## PARP Inhibitors (PARPi)

Currently, four agents (olaparib, rucaparib, niraparib, and talazoparib) are approved for the treatment of different tumors, including ovarian, breast, prostate, and pancreatic cancers. The success of PARPi in cancer treatment is believed to originate from their ability to induce synthetic lethality (27, 28). Synthetic lethality arises when the co-occurrence of two gene conditions causes cell death, whereas a deficiency in only one of the genes does not determine cell lethality (29). Mechanistically, PARPi anticancer agents compete with nicotinamide (NAD^+^) for the PARP catalytic site, inhibiting single-strand break repair (8, 30, 31). This effect promotes the accumulation of single-strand breaks that result in the collapse of the replication fork and replication associated with the double-strand break. Subsequently, tumor cells become more dependent on the HR or NHEJ repair pathways (18, 19). In tumors with HR defects, such as those with *BRCA1/2* mutations, PARPi induces synthetic lethality*. BRCA2*-deficient cells compared to *BRCA2*-proficient cells are 90 times more sensitive to PARP inhibition (18, 32). Although there is a consensus that PARPi mechanisms of action rely on inducing synthetic lethality in tumors with defective HR, more recent findings suggest that PARPi also modulates the antitumor immune response.

## Immune Effects of PARP Inhibitors

Previous reports have noted an association between DDR defects or failure with the activation of anticancer immunity through the response-dependent type I interferon (IFN) pathway or *via* the accumulation of mutations and neoantigens (33–35). In this context, novel findings have demonstrated that the pharmacological inhibition of PARP can mimic this condition, dramatically affecting the balance of the immune response in the tumor microenvironment.

### The DNA Damage Induced by PARPi Induces Antitumor Immune Response

DNA sensing through the cyclic guanosine monophosphate–adenosine monophosphate synthase (cGAS)/stimulator of interferon genes (STING) pathway participates in host defense by detecting aberrant entry of DNA into the cytosol (36). This pathway is classically involved in defense against viruses; however, new evidence indicates that the cGAS-STING pathway is also activated by fragments of endogenous DNA generated by cancer treatment, driving an effective antitumor immune response (36–38).

In preclinical studies, PARPi effectiveness in *BRCA1*-deficient tumors was found to be dependent on CD8 T-cell recruitment *via* intratumoral cGAS/STING pathway activation. The use of PARPi in DRR-defective tumors produces single-and double-strand breaks in DNA that bind to cGAS, leading to the production of a second messenger molecule that stimulates the adapter protein STING. STING, *via* kinases TANK-binding kinase 1 (TBK1) and IkappaB kinase (IKK), activates transcription factor interferon regulatory transcription factor 3 (IRF3) and factor nuclear kappa B (NF-κB), which translocate into the nucleus to trigger type I IFN signaling (39).

It is well known that IFNs play a central role in antitumor immunity (40). The seminal demonstration that interferon-α/β receptor (IFNAR) or signal transducer and activator of transcription 1 (STAT1) knockout mice fail to reject immunogenic tumors (41, 42). Numerous studies have shown that the expression levels of IFNs are positively correlated with CD8 + T cell lymphocyte infiltration in the tumor microenvironment (39, 43, 44). Thus, the introduction of DNA damage by PARPi can trigger the transformation of tumors from cold to hot (39). Moreover, CD8 T lymphocytes kill malignant cells upon recognition by the T-cell receptor (TCR) of specific antigenic peptides present on the surface of the target cells (45). In this context, effective antitumor immunity relies on cross-presentation of tumor antigens by APCs to CD8 T lymphocytes. APC activation requires type I IFN signaling, which can be initiated by cGAS-STING activation (40, 42, 46, 47). Therefore, cGAS-STING signaling can act as a bridge between DNA damage and the activation of anticancer immune responses.

However, in parallel, type I IFNs activate pathways that control the exacerbated inflammatory immune responses. For example, IFN-β has been shown to induce the expression of PD-L1 in tumor cells, which contributes to the immune escape by cancer cells (48). In line with our premise, PARPi induces upregulation of PD-L1 in tumor cells (49).

### The Genomic Instability Induced by PARPi Triggers Antitumor Immune Response

In tumor cells, DDR failure can result in the accumulation of mutations in drive genes that produce survival advantages and accelerate tumor development (50). However, this genomic instability can encode tumor-specific neoantigens, which may make tumors more attractive to the immune response (51, 52). There is a correlation between tumor mutational burden and the likelihood of response to ICIs. Preclinical studies have shown that cancer cells with microsatellite instability (MSI) or defective mismatch repair (dMMR) grow in immune-deficient mice but are unable to grow in immune-competent mice (53). In clinics, MSI or dMMR are biomarkers for predicting responses to ICIs approved by the FDA (54). In this context, it has been discussed whether drugs that modulate DDR pathways, such as PARPis, can promote genetic instability and neoantigen formation.

It has been experimentally demonstrated that in *BRCA*-deficient cells, PARPi induces chromosomal instability typified by the accumulation of chromosomal breaks and eventual lethality *via* NHEJ (24). In another study, genomic instability and cell death induced in *BRCA1*-deficient cells by PARPi were found to be dependent on the NHEJ factor p53-binding protein 1 (53BP1) (55). Although this mechanism still needs to be further explored clinically, these primary findings suggest that the pharmacological blockade of PARP has the potential to increase genomic instability and lead to dynamic mutational profiles, resulting in the persistent renewal of neoantigens and engagements of an immune response.

## The Rationale for the Combination of ICIs and PARPi

Immune checkpoints represent a set of modulatory pathways essential for exacerbating inflammatory responses and maintaining self-tolerance (56). The receptors cytotoxic T-lymphocyte-associated protein 4 (CTLA-4) and PD-1, expressed mainly in lymphocytes, and PD-L1, expressed in APCs, are part of the immunological checkpoint system (57). The interaction between CTLA-4 and CD80, CD86, or PD-L1/PD-1 reduces T cell activity, leading to suppression of the inflammatory response and preservation of tissues (57, 58). However, this mechanism favors cancer progression, enabling the escape of the anti-tumor immune response. Therefore, the use of monoclonal antibodies (mAbs) to block CTLA-4, PD-1, or its ligand PD-L1, ICIs, reactivates and drives the immune response to detect and destroy tumors by overcoming the negative feedback mechanism of the immune response (59).

Although there is no doubt that ICI therapy positively impacts cancer treatment in several neoplasms, ICIs may not be sufficient for optimal antitumor activity in some patients, particularly those with a defect in cancer antigen-specific T-cell activation or impairment of T-cell infiltration into tumors (60). Thus, efforts to enhance these responses are needed. The interaction between tumor DNA damage and the immune system plays a role in driving the response to ICI. DNA-damaging agents include chemotherapy (CT), ionizing radiation (RDT), and targeted DNA repair therapies. CT activates the immune system by inducing immunogenic cell death pathways. RT causes several types of DNA damage. DNA repair targeted agents include PARPi. In particular, combination strategies with PARPi can potentially maximize the benefit from ICIs, and its plausible synergistic effect resides in the immune properties of PARPi at different points in the cancer immune response. PARPi may facilitate a more profound antitumor immune response and synergize with ICIs by inducing DNA damage, producing a T helper 1 (Th1) immune-mediated response *via* IFN signaling, activation of APC cells, increased recruitment of effector lymphocytes, and promoting upregulation of PD-L1 in tumor cells (39, 49, 61).

**Figure 1:**
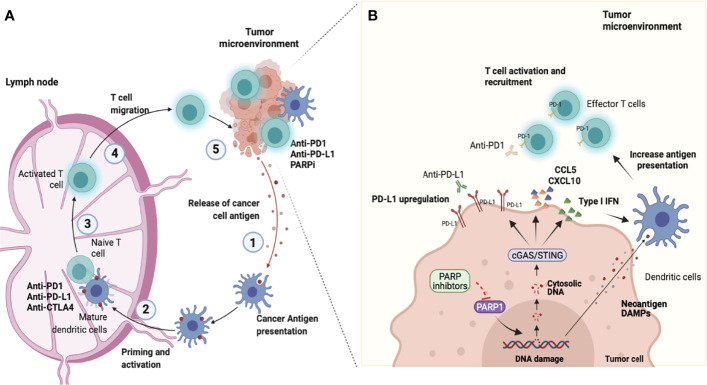
Combining PARP inhibition and immune checkpoint blockade. **(A)** Antitumor immunity depends on a series of stepwise events. Primarily this process includes the capture and processing of Tumor-associated antigens (TAAs) by Antigen-presenting cells (APCs), such as dendritic cells or macrophages in the tumor microenvironment (step 1). Next, APCs cells presented antigen to CD8+ T cells at the lymph nodes (step 2). This process promotes the prime and activation of effector CD8 T cells (step 3). Finally, the activated effector T cells migrate from lymphocytes (step 4) and infiltrate into the tumor microenvironment to recognize and eliminate tumor cells (step 5), completing the cancer-immune cycle. However, the continued immune attack may enable cancer cells to evolve mechanisms for the escape of immune attacks. Molecules that negatively regulate T lymphocyte activation, called immune checkpoints are central players involved in tumor immune escape. In the cancer-immune cell cycle the ICIs (anti-CTLA-4, anti-PD-1, or anti-PD-L1) reactivate and drive the immune response to detect and destroy tumors by overcoming the negative feedback mechanism of the immune response acting in steps 3 and 5. **(B)** Poly-ADP-ribose polymerase inhibitors (PARPi) have effects in the early steps of the cancer-immune cell cycle. PARPi induce DNA breaks in *BRCA1/2*-deficient cells which can result in cell death or genomic instability and neoantigen formation. Furthermore, the DNA damage induces the release of DNA fragments into the cytosol which causes the cGAS/STING pathway activation in tumor cells and the production of type I IFN and chemokines (CCL5 and CXCL10). This effect culminates with paracrine activation of APCs such as dendritic cells (step 1 and 2) and with the recruitment of CD8 cells for the tumor microenvironment (step 4). Another important immune effect of PARPi is associated with the increased expression of Programmed death ligand-1 (PD-L1) in tumor cells (step 5). Therefore, the combined use of PARPi with Immune checkpoint inhibitors (ICIs) has the potential to amplify the entire cancer immune cycle (image created at Biorender).

In summary, DNA damage induced by a PARPi can promote antitumor immunity *via* the cGAS-STING/type I IFN/CD8 army (positive effect). In contrast, type I IFN induces PD-L1 expression and promotes tumor immune escape (a negative effect). In this context, the combination of PARPi and ICIs has particular translational appeal owing to its potent immune-stimulatory anticancer effects (**Figure 1**).

## PARPi and ICIs Combination in Preclinical Studies

In preclinical models, PARPi has demonstrated synergy with ICIs in a variety of tumor models regardless of *BRCA1/2*-defect. It was demonstrated that PARPi-based therapy synergizes with anti-PD-1 against both MSI and microsatellite stable (MSS) colon cancer models, with a potential sensitizing effect of anti-PD-1 therapy against MSS tumors (61). In another study, PARPi led to the accumulation of cytosolic double-stranded DNA, thereby activating type I IFN-related immune response. Shen et al. (2019) (62) demonstrated the combined use of PARPi and ICIs against colon and ovarian experimental tumors, regardless of the *BRCA1/2* mutation status of the cell lines assessed both *in vitro* and *in vivo*. Furthermore, PARPi treatment upregulated PD-L1 expression *in vitro* and *in vivo* in breast cancer cell lines, xenograft tumors, and syngeneic tumors. Although PARPi attenuated anticancer immunity *via* upregulation of PD-L1, the combination of PARPi and anti-PD-L1 therapy compared with each agent alone significantly increased therapeutic efficacy (49). Investigating the effects of the PARP1/2 inhibitor niraparib in combination with ICI therapy in *BRCA*-deficient and BRCA-proficient breast cancer tumor models, it was observed that the combined regimen demonstrated synergistic antitumor activity in both *BRCA*-proficient and *BRCA*-deficient tumors. Interestingly, mice with tumors cured by single-agent niraparib completely rejected tumor growth upon rechallenge with the same tumor cell line, suggesting the potential establishment of immune memory (63).

Together, these data reinforce that PARPi in combination with ICIs may be beneficial in tumors, regardless of DNA repair status, which has important clinical implications.

## PARPi and ICIs Combination in Clinical Studies

### Combination of a PARPi With Anti-PD1/PD-L1 ICIs: What Do We Already Know?

Phase I study analysis of this combination showed toxicities manageable with supportive care, and no new adverse events were noted compared with the PARPi or ICI toxicities in monotherapy (64, 65) A phase I study of solid tumors tested a combination of durvalumab, an anti-PD-L1 agent, and olaparib. Durvalumab was administered at 10 mg/kg every 2 weeks or 1,500 mg every 4 weeks, and olaparib tablets were administered twice daily. No dose-limiting toxicity was observed for durvalumab plus olaparib. Two partial responses (≥15 months and ≥ 11 months) and eight stable diseases ≥ 4 months (median, 8 months [4–14.5 months]) were seen in patients who received this combination, generating an 83% disease control rate (65).

Here, we explored clinical trials evaluating the efficacy of PARPi and anti-PD1/PD-L1 ICIs in ovarian and breast cancers. Studies with breast cancer and ovarian cancer patients, as summarized in **Table 1**, demonstrate interesting response rates with acceptable toxicity.

**Table 1:** Clinical trials evaluating the combination of PARP inhibitors and immune checkpoint inhibitors in breast cancer ovarian cancer.

Studies in Breast Cancer	Immunotherapy	PARPi	Patients	Outcome
NCT02657889 (TOPACIO/KEYNOTE-162) Phase II	Pembrolizumab (200 mg Q3W)	Niraparib (200 mg QD)	**N=55** Advanced/Metastatic TNBC	ORR 21% with 5 CRs and 5 PRs (better *BRCA*-mutated tumors), DCR 49%
NCT02734004 (MEDIOLA) Phase II	Durvalumab (1500 mg Q4W)	Olaparib (300 mg BID)	**N=34** *gBRCAm* HER2 negative mBC	28-week DCR 47%, ORR 56%, PFS 6.7 months.
NCT03330405 (JAVELIN PARP Medley) Phase Ib/II	Avelumab (800 mg Q2W)	Talazoparib (1mg QD)	**N=34** Previously Treated advanced solid tumors	First-cycle DLT 25% ORR 8% with 1 PR, SD 50%
**Studies in ovarian cancer**				
NCT02571725 Phase I	Tremelimumab (10 mg/kg Q4W)	Olaparib (300 mg BID)	**N=3** *gBRCAm* recurrent ovarian cancer	No DLT or grade 3 AE ORR 100% with 3 PRs
NCT02484404 Phase II	Durvalumab (1500 mg Q4W)	Olaparib (300 mg BID)	**N=35** Platinum-resistant recurrent ovarian cancer	ORR 14% with 5 PRs, DCR 71%, mPFS 3.9 months
NCT02657889 (TOPACIO/KEYNOTE-162 Phase II	Pembrolizumab (200 mg Q3W)	Niraparib (200 mg QD)	**N=60** Platinum-resistant recurrent ovarian cancer	ORR 18% with 3 CRs and 8 PRs (irrespective of *BRCA* and HRD status), DCR 65% mPFS 3.4 months
NCT02734004 (MEDIOLA) Phase II	Durvalumab (1500 mg Q4W)	Olaparib (300 mg BID)	**N=32** *gBRCAm* platinum-sensitive ovarian cancer	12-week DCR 81%, ORR 63% with 6 CRs and 14 PRs
NCT02660034 Phase I	Tislelizumab (200 mg q3W)	Pamiparib (40mg BID)	**N=49** advanced and previously treated solid tumors	ORR 20%. RP2D

TNBC, triple-negative breast cancer; gBRCAm, germline breast cancer gene mutation; BRCA, breast cancer gene; N, number of patients; ORR, overall response rate; CR, complete response; PR, partial response; SD, stable disease; DCR, disease control rate; PFS, progression-free survival; HRD, homologous recombination deficiency; DLT, dose-limiting toxicities. RP2D: recommended phase 2 dose. QD, daily; BID, two times per day; Q2W, 2 week cycle; Q3W,, 3 week cycle; Q4W, 4 week cycle.

MEDIOLA is a phase II basket study assessing the efficacy and safety of a chemo-free combination of olaparib and durvalumab in patients with solid tumors (NCT02734004) and germline *BRCA1/2* (*gBRCA1/2*) mutations. Patients received olaparib for 4 weeks, followed by a combination of olaparib and durvalumab until disease progression. The primary endpoints were the disease control rate (DCR) at 12 weeks, safety, and tolerability. Patients with platinum-sensitive recurrent ovarian cancer (n=34) received at least one prior line of platinum therapy. The 28-week DCR was 65.6%, while the overall response rate (ORR) was 71.9%, with a total of seven complete responses (CRs). The median progression-free survival (PFS) was 11.1 months (95% CI: 8.2, 15.9), with a median duration of response (DOR) of 10.2 months. The median overall survival (OS) for all patients is not yet reached, with 87.0% of patients alive at 24 months (66). Thirty-four patients were enrolled in the human epidermal growth factor 2 receptor (HER2)-negative metastatic breast cancer group. The 12- and 28-week DCRs were 81% and 47%, respectively. The ORR for the overall cohort was 56%, with one patient with CR and six (19%) patients with progressive disease (PD). The median PFS was 6.7 months (95% CI: 4.6, 11.7 months). The most common grade 3 or 4 adverse events reported were anemia (11.8%), neutropenia (8.8%), and pancreatitis (5.9%) (67). Therefore, we concluded that the combination of olaparib and durvalumab was well tolerated and showed promising median PFS and DOR for ovarian cancer, breast cancer, and gBRCA1/2 mutations.

The TOPACIO/KEYNOTE-162 phase I/II study evaluated the efficacy and safety of nivolumab plus pembrolizumab in platinum-resistant recurrent ovarian cancer and metastatic triple-negative breast cancer (TNBC). This trial included patients with or without gBRCA mutations. The primary outcome was ORR. In the ovarian cancer group (n = 60), ORR 18% with 3 CRs and 8 PRs (irrespective of *BRCA* and HRD status), and DCR 65%. The median PFS was 3.4 months, with acceptable toxicity. Responses in patients without tumor *BRCA* mutations were higher than expected with either agent as monotherapy (68). Of 46 breast cancer evaluable patients, 20 (49%) achieved durable clinical benefit (any complete response/partial response or stable disease ≥16 weeks), with stronger activity in BRCA-mutated tumors (69).

The PARPi talazoparib was also evaluated in the phase Ib/II study. Patients with advanced solid tumors who had received ≥1 prior standard of care chemotherapy regimen were treated with Avelumab in combination with Talazoparib. In phase 2 cohorts, eligible patients had metastatic TNBC (cohort 2A) or hormone receptor-positive (HR+), HER2 negative, DNA damage repair defect-positive breast cancer (cohort 2B). Patients in cohort 2A/B had received 0 to 2 prior therapies (no progression on prior platinum-based chemotherapy). The primary endpoint was the objective response. A total of 22 patients had been treated in both cohorts. In cohort 2A, 12 patients were evaluable for disease assessment: PR in 1, SD in 6, and PD in 5. All 3 patients in cohort 2B were non-evaluable for response at data cutoff. Treatment-related Adverse events (AEs) of any grade occurred in 94.7% of patients, the most common AEs were anemia, nausea, fatigue, and thrombocytopenia; 9 patients (47.4%) had grade ≥3 AEs. Therefore, Avelumab administered in combination with Talazoparib in patients with advanced solid tumors showed preliminary antitumor activity and a manageable safety profile. The study is ongoing (70).

Michael Friedlander and colleagues reported the findings of a phase 1a/b trial of the combination of a PARPi (Pamiparib) and ICI (Tislelizumab) in 49 patients with previously treated, advanced solid tumors. The results from the dose-escalation stage, phase 1a/b trial, show that the combination was generally well tolerated and associated with antitumor responses (20%) in patients with advanced solid tumors supporting further investigation of the combination (64).

### Combination of PARPi With ICIs: Ongoing Studies

There are numerous ongoing trials (phases I-III) exploring the combination of PARPi and anti-PD1/anti-PD-L1 agents, and some trials with new immunotherapy agents such as TSR-022. TSR-022 is a monoclonal antibody against T-cell immunoglobulin and mucin domain molecule 3 (TIM-3) (also called HAVCR2), an immune checkpoint receptor. **Table 2** summarizes the ongoing phase III studies with a combination of immunotherapy and PARPi.

**Table 2:** Ongoing studies with a combination of immunotherapy and PARP inhibitors.

Ongoing Phase III Studies	Immunotherapy	PARPi Agent	Patients	Outcome
NCT03740165 (KEYLYNK-001)	Pembrolizumab + CT	Olaparib (maintenance)	First-Line Treatment of Women with *BRCA* Non-mutated Advanced Ovarian Cancer	PFS
NCT04191135 (KEYLYNK-009)	Pembrolizumab	Olaparib	First-Line in Triple Negative Breast Cancer after induction CT + embrolizumabe	PFS
NCT03737643 (DUO-O)	Durvalumab +/- Bevacizumab	Olaparib (maintenance)	Newly diagnosed advanced ovarian, fallopian tube or primary peritoneal carcinoma or carcinosarcoma	PFS
NCT03598270 (ANITA)	Atezolizumab + Platinum-based Chemotherapy	Niraparib	Patients with Recurrent Ovarian Cancer	PFS
NCT03522246 (ATHENA)	Nivolumab	Rucaparib	Maintenance Treatment Following Response to Front-Line Platinum-Based Chemotherapy in Ovarian Cancer Patients	PFS
NCT03642132 (JAVELIN OVARIAN PARP 100)	Avelumab	Talazoparib	Maintenance therapy in Untreated Advanced Ovarian Cancer patients	PFS
NCT03602859 (FIRST)	Platinum-based Therapy With TSR-042	Niraparib	First-line Treatment of Stage III or IV Nonmucinous Epithelial Ovarian Cancer	PFS
**Ongoing phase I/II trials**				
NCT03101280	Atezolizumab	Rucaparib	Participants with Advanced Gynecologic Cancers and TNBC	AE; DLTs Recommended Dose of Rucaparib3.
NCT02849496	Atezolizumab	Olaparib	*BRCA* Mutant Non-HER2- Locally Advanced or Metastatic Breast Cancer	PFS; ORR
NCT03307785	TSR-022 & TSR-042	Niraparib	Patients with Advanced or Metastatic Cancer	DLT; AE; ORR3
NCT03565991 (Javelin BRCA/ATM)	Avelumab	Talazoparib	Patients with *BRCA* or ATM Mutant metastatic Solid Tumors	OR; TTR; DOR; PFS; OS
NCT02660034	Tislelizumab	Pamiparib	Subjects with Advanced Solid Tumors	AE; DLT; ORR; PFS; DOR; OS
NCT02484404	Durvalumab	Olaparib and/or Cediranib	Advanced Solid Tumors and Advanced or Recurrent Ovarian, Triple Negative Breast, Lung, Prostate and Colorectal Cancers	ORR; RP2D

CT, chemotherapy; BRCA, breast cancer gene; TNBC, triple-negative breast cancer; ATM, ataxia telangiectasia mutated; HER2, human epidermal growth factor 2 receptor; AE, adverse events; PSF, progression-free survival; ORR, overall response rate; DOR, duration of response; OR, objective response; TTR, time to tumor response; DLT, dose-limiting toxicities; RP2D, recommended phase 2 dose.

The association between PARPi and anti-CTLA-4 has been less studied. The combination of PARPi and CTLA-4 blockade is tolerable in heavily pretreated women with recurrent *BRCA*-associated ovarian cancer (62). Preliminary results of a phase I study combining olaparib and tremelimumab demonstrated evidence of therapeutic effects, supporting the ongoing evaluation of this regimen in phase II trials: NCT02571725 (71).

### Targeting DNA Damage Signaling Proteins: Beyond PARP Inhibitors

The mechanisms that have inspired numerous PARPi-based combination therapies, including immunotherapy, also capitalize on the potential synergistic effects of different inhibitors of the DDR pathway, such as ataxia telangiectasia and Rad3-related (ATR), ataxia telangiectasia mutated (ATM), and Checkpoint kinase 1 (CHK1) inhibitors.

Preclinical data have demonstrated a synthetic lethal interaction between ATR and the ATM-p53 pathway in cells that respond to DNA damage. In a large proportion of cancer cells, where ATM-p53 signaling is defective, initiation of DNA replication continues and DNA damage accumulates, leading to cell death (72). It was demonstrated that combined treatment with ATR and CHK1 inhibitors leads to replication fork arrest, single-stranded DNA accumulation, replication collapse, and synergistic cell death in cancer cells *in vitro* and *in vivo* (73).

Strikingly, in addition to direct cytotoxic effects, ATM, ATR, and CHK1/2 inhibitors potentiate antitumor immunity. Inhibition of ATM/Chk2 led to replication stress and accumulation of cytosolic DNA, which subsequently activated the STING-mediated immune response (74). Vendetii et al. (2018) and Sheng et al. (2018), the ATR kinase inhibitor AZD6738 combined with radiation therapy boosted infiltration, increased cell proliferation, enhanced IFNγ production by CD8 T cells, and caused a decrease in the number of Tregs and exhausted T cells in the tumor in mouse models. Mechanistically, this study revealed that the antitumor effect of AZD6738 relied on the activation of the cGAS/STING pathway. These findings indicate that inhibitors of key DRR mechanisms, beyond PARP, promote the antitumor immune response through activation of the STING pathway (75, 76).

The proposed rational approach to enhance the efficacy of ICIs to utilize DRR inhibitors, to increase tumor DNA damage and thereby ‘prime’ tumors for response to immune ICIs have been explored in mouse models. The genic deletion of ATM induced IFN response and enhanced lymphocyte infiltration into the tumor microenvironment *via* cGAS/STING activation. This effect potentiated ICI therapy in mouse melanoma (B16) and breast cancer (4T1) tumors (77). In another study, tumor immunogenicity was evaluated after the pharmacological inhibition of ATM following PD-L1/PD-1 checkpoint inhibition. ATM inhibition increased the tumoral expression of type-I IFN in a TBK1- and SRC-dependent manner. Furthermore, ATM silencing increased PD-L1 expression, tumoral CD8 cells, and the sensitivity of pancreatic tumors to ICIs, suggesting that the efficacy of ICIs in pancreatic cancer can be enhanced by ATM inhibition (78). Similarly, ATM inhibition in tumors with a mutation in AT-rich interactive domain-containing protein 1A (ARID1A), a component of the chromatin-remodeling complex switch/sucrose-nonfermentable (SWI/SNF), selectively potentiates replication stress and accumulation of cytosolic DNA, which subsequently activates the DNA sensor STING-mediated innate immune response in ARID1A-deficient tumors. In patients, tumors with mutations or low expression of both ARID1A and ATM/CHK2 exhibit increased tumor-infiltrating lymphocytes and are associated with longer patient survival (74).

The combination of SRA737, an oral CHK1 inhibitor, with or without anti–PD-L1/anti-PD-1 leads to an antitumor response in multiple cancer models, including Small Cells Lung Cancer (SCLC). The combination of low-dose non-cytotoxic gemcitabine with SRA737 plus anti–PD-L1 increased the expression of type I IFN genes and chemokines (CCL5 and CXCL10*)*, and the number of CD8, dendritic cells, and M1 macrophages in the tumor microenvironment. Using the PARPi (olaparib) or the CHK1 inhibitor (prexasertib) in combination with anti-PD-L1, a significant increase in cytotoxic T-cell infiltration inducing tumor regression was observed in the SCLC mouse model (79). Mechanistically, it was demonstrated that the treatment with DDR inhibitors activated the STING/TBK1/IRF3 pathway, leading to increased levels of chemokines (CXCL10 and CCL5), which recruited and activated CD8 T lymphocytes into the tumors (80).

In the clinics the ATR inhibitor ceralasertib has been tested in phase I in combination with chemotherapy, olaparib, or an anti-PD-L1 antibody. The durvalumab plus ceralasertib combination arm enrolled 25 patients with advanced head and neck squamous cell carcinoma or non-small cell lung cancer (NSCLC). The primary objective was to recommend a phase 2 dose of ceralasertib. Of the 21 patients evaluated, one complete response and three partial responses were observed, independent of tumor PD-L1 expression. They concluded that this combination is tolerated in dose escalation, with preliminary signals of antitumor activity in patients with advanced solid tumors (81). Berzosertib, another ATR inhibitor, has been tested in a phase IB/II study of combination chemotherapy and pembrolizumab in patients with advanced NSCLC with squamous cell histology; the estimated enrollment was 18 participants (NCT04216316).

## Conclusion and Perspectives

The combination of PARPi and ICIs is promising and has been explored in various clinical trials. While most studies with this combination have focused on patients with ovarian or breast cancer harboring germline pathogenic variants in *BRCA1/2* genes, other tumor histologies, including prostate cancer and pancreatic cancer, have been studied (82). Biomarkers trying to identify patients whose tumors have HR defects without germline *BRCA* mutations that could benefit from this combinatorial approach have also been explored. The results of ongoing phase III studies are awaited and can change the landscape of treatment for these patients.

## Authors Contributions

RB-S conceived the work. CW, TC, FC, MS, and RB-S wrote the manuscript. All authors contributed to the article and approved the submitted version.

## Funding

Instituto de Pesquisa e Ensino (IEP) - Hospital Sírio Libanês and Fundação de Amparo à Pesquisa do Estado de São Paulo - FAPESP (2017/25308-9).

## Author Disclaimer

All claims expressed in this article are solely those of the authors and do not necessarily represent those of their affiliated organizations, or those of the publisher, the editors, and the reviewers. Any product that may be evaluated in this article, or claim that may be made by its manufacturer, is not guaranteed or endorsed by the publisher.

## Conflict of Interest

The authors declare that the research was conducted in the absence of any commercial or financial relationships that could be construed as a potential conflict of interest.

The handling Editor declared a shared affiliation with the authors CW and FC at the time of review.

## Publisher’s Note

All claims expressed in this article are solely those of the authors and do not necessarily represent those of their affiliated organizations, or those of the publisher, the editors and the reviewers. Any product that may be evaluated in this article, or claim that may be made by its manufacturer, is not guaranteed or endorsed by the publisher.

## Abbreviations

53BP1, p53-binding protein 1; AEs, Adverse events; APCs, Antigen present cells; ARID1A, AT-rich interactive domain-containing protein 1A; ATM, Ataxia telangiectasia mutated; ATR, Ataxia Telangiectasia Rad3-related; BC, Breast cancer; BRCA, Breast cancer gene; BRCA1, Breast cancer gene 1; BRCA2, Breast cancer gene 2; cGAS, Cyclic guanosine monophosphate–adenosine monophosphate synthase; CHK1, Checkpoint kinase1; CHK2, Checkpoint kinase2; CRs, Complete responses; CT, Chemotherapy; CTLA-4, Cytotoxic T-lymphocyte-associated protein 4; DCR, Disease control Rate; DDR, DNA damage response; DLT, Dose-limiting toxicities; dMMR, Defective mismatch repair; DNA-PKcs, DNA-dependent protein kinase; DOR, Duration of response; HER2, Human epidermal growth factor 2 receptor; HR, Homologous recombination; ICIs, Immune checkpoint inhibitors; IFNAR, Interferon-α/β receptor; IFNs, type I interferons; IKK, IkappaB kinase; IRF3, Interferon regulatory transcription factor 3; Ku70/80, Ku heterodimer protein; MRE11, Meiotic recombination 11; MSI, Microsatellite instability; MSS, Microsatellite stable; NAD+, Nicotinamide; NBS1, Nijmegen breakage syndrome 1; NF-κB, Factor nuclear kappa B; NHEJ, Non-homologous end-joining; OC, Ovarian cancer; OR, Objective Response; ORR, Overall response rate; OS, Overall survival; PARP, Poly (ADP-ribose) polymerases; PARP1, Poly (ADP-ribose) polymerase 1; PARPi, Poly-ADP-ribose polymerase inhibitors; PD, Progressive disease; PD-1, Receptor of programed death 1; PD-L1, Programmed death ligand-1; PFS, Progression-free survival; PR, Partial response; RAD50, RAD50 homolog; RPA, Replication protein A; SCLC, Small Cells Lung Cancer; SD, Stable disease; SOC, Serous ovarian cancers; STAT1, Signal transducer and activator of transcription 1; STING, Stimulator of interferon genes; SWI/SNF, Switch/Sucrose-Nonfermentable; TAAs, Tumor-associated antigens; TBK1, TANK-binding kinase 1; TCR, T-cell receptor; TFAM, Mitochondrial transcription factor A; Th1, T helper 1; TILs, Tumor-infiltrating lymphocytes; TTR, Time to tumor response; XLF, XRCC4-like factor; XRCC1, X-Ray Repair Cross Complementing 1; XRCC4, X-ray cross-complementing protein 4.
